# Type of Anion Largely Determines Salinity Tolerance in Four *Rumex* Species

**DOI:** 10.3390/plants12010092

**Published:** 2022-12-24

**Authors:** Zaiga Landorfa-Svalbe, Una Andersone-Ozola, Gederts Ievinsh

**Affiliations:** Department of Plant Physiology, Faculty of Biology, University of Latvia, 1 Jelgavas Str., LV-1004 Riga, Latvia

**Keywords:** chloride, coastal plants, dock species, electrolyte accumulation, halophytes, ion accumulation, nitrate, nitrite, salinity tolerance, water content

## Abstract

The aim of the present study was to compare the effect of various salts composed of different cations (Na^+^, K^+^) and anions (chloride, nitrate, nitrite) on growth, development and ion accumulation in three *Rumex* species with accessions from sea coast habitats (*Rumex hydrolapathum, Rumex longifolius* and *Rumex maritimus*) and *Rumex confertus* from an inland habitat. Plants were cultivated in soil in an experimental automated greenhouse during the autumn–winter season. Nitrite salts strongly inhibited growth of all *Rumex* species, but *R. maritimus* was the least sensitive. Negative effects of chloride salts were rather little-pronounced, but nitrates resulted in significant growth stimulation, plant growth and development. Effects of Na^+^ and K^+^ at the morphological level were relatively similar, but treatment with K^+^ salts resulted in both higher tissue electrolyte levels and proportion of senescent leaves, especially for chloride salts. Increases in tissue water content in leaves were associated with anion type, and were most pronounced in nitrate-treated plants, resulting in dilution of electrolyte concentration. At the morphological level, salinity responses of *R. confertus* and *R. hydrolapathum* were similar, but at the developmental and physiological level, *R. hydrolapathum* and *R. maritimus* showed more similar salinity effects. In conclusion, the salinity tolerance of all coastal *Rumex* species was high, but the inland species *R. confertus* was the least tolerant to salinity. Similarity in effects between Na^+^ and K^+^ could be related to the fact that surplus Na^+^ and K^+^ has similar fate (including mechanisms of uptake, translocation and compartmentation) in relatively salt-tolerant species. However, differences between various anions are most likely related to differences in physiological functions and metabolic fate of particular ions.

## 1. Introduction

Soil salinity is one of the global problems for crop production in virtually all regions of the world; therefore, there is an increasing practical interest in understanding salinity tolerance mechanisms of plants. Wild plants from natural salt-affected habitats represent a useful resource in salinity tolerance studies [1,2]. In this respect, coastal habitats are an especially promising and underexploited environment, mostly due to the fact that not only coastal-specific plant species occur in coastal regions, and general-distribution species from salt-affected coastal soils are of great interest [3,4]. On the other hand, understanding the coexistence of species with different salt tolerance strategies is important for understanding of vegetation zonation in coastal habitats [5].

So far, in salinity tolerance studies, attention has mainly been focused on the effects of NaCl, with special attention paid to “Na^+^ toxicity” [6]. In this respect, general physiological strategies of interest for adaptation to saline heterogeneous environments have been studies of Na^+^ exclusion and accumulation as well as mechanisms for ensuring sufficient K^+^ levels. However, in many situations, other ions besides Na^+^ and Cl^−^ participate in the formation of salinity [7]. Therefore, for both practical and theoretical reasons, studies comparing plant responses to salinity caused by various salts consisting of different cation and anion components are of critical importance for achieving a broader understanding of salinity tolerance.

An ability of Na^+^ to substitute K^+^ has been suggested as an important feature of salinity tolerance in halophytic species [8]. The similar responses of several halophytic plant species to Na^+^ and K^+^ in a chloride form have been indicated previously [8,9,10]. Recently, a number of relatively salt-tolerant species from coastal habitats showed identical responses to NaCl and KCl treatment, including *Armeria maritima* [11], *Beta vulgaris* subsp. *maritima*, *Cochlearia officinalis*, *Mentha aquatica* and *Plantago maritima* [12]. The same type of response was also characteristic for a cultivated crop taxon, *Beta vulgaris* subsp. *vulgaris* var. *cicla* [12]. It was concluded that mainly the anionic component of salts could determine the nature of salinity response in coastal salinity-adapted species.

One less-explored aspect in salinity studies is the effect of mineral availability and mineral nutrition on salinity tolerance and ion accumulation. Differences in N availability are shown to modulate NaCl effects in *Solanum melongena* [13], *Suaeda salsa* [14] and *Suaeda physophora* [15]. In contrast to the relatively large number of studies on the effect of alkaline salts (NaHCO_3_, Na_2_CO_3_) [16], N-containing salts have been rarely used in plant salinity tolerance studies [10,17].

The genus *Rumex* represents species with contrasting adaptations to soil moisture. While there are several *Rumex* species characteristically found in dry habitats (i.a., *Rumex acetosella*, *Rumex thyrsiflorus*), a group of wetland *Rumex* species is well-adapted to soil waterlogging and flooding [18]. Some of these species have been used as models in flooding tolerance studies (i.a., *Rumex maritimus* and *Rumex palustris*) [19]. Several *Rumex* species frequently occur in sea-affected habitats. In a coastal seawater-affected marsh, plants of *Rumex hydrolapathum*—an emergent macrophyte from flooded or permanently wet habitats [20]—showed high potential to accumulate both Na^+^ and K^+^ in leaves at levels significantly higher than those of adjacent plants [3]. This accession also displayed a high tolerance against biogenous heavy metals Zn and Mn, and extremely high metal accumulation potential in controlled conditions [21]. In the present study, three *Rumex* species with accessions from sea coast habitats (*Rumex hydrolapathum, Rumex longifolius* and *Rumex maritimus*) were chosen for salinity experiments in controlled conditions, and were compared to *Rumex confertus*, a cosmopolitan species not occurring in saline soils [22]. None of these *Rumex* species are described as halophytic or salt-tolerant, with respective Ellenberg indicator values for salinity being 0 [23]. However, according to the Swedish ecological indicator values, *R. confertus*, *R. hydrolapathum* and *R. longifolius* have salinity tolerance levels of 2 out of 5, meaning that these species are “moderately salt-tolerant, but preferring non-saline conditions” [24]. Only *R. maritimus* is characterized as “favoured by moderate salinity, but not restricted to such habitats” (tolerance level 3). In respect to nitrogen availability, *R. confertus*, *R. hydrolapathum* and *R. maritimus* are characterized as “confined to the naturally most N-rich soils” (index value 8 out of 9), but *R. longifolius* is found in very N-rich habitats (index value 7) [24]. However, moisture requirements of the four species are relatively variable, with their habitats being characterized as mesic (4 out of 12; *R. longifolius*), moist–wet (7; *R. confertus* and *R. maritimus*) and wet–temporarily inundated (9; *R. hydrolapathum*) [24].

The aim of the present study was to provide experimental evidence for the importance of anionic components in salinity responses to Na^+^ and K^+^ at the level of both growth and ion accumulation with an emphasis on possible anion toxicity. Na^+^ and K^+^ salts were in the form of nitrates, necessary for all plants as a source of N [25]; chlorides, possibly beneficial for halophytes at low concentrations [26]; and nitrites, possibly toxic for all plant species [27]. It was proposed that accessions of the three *Rumex* species from coastal habitats would be better adapted to salinity and will have a larger electrolyte accumulation capacity for both Na^+^ and K^+^ in comparison to that of the non-coastal species *R. confertus*. Due to possible changes in tissue water content, ion accumulation patterns in various plant parts were assessed both on dry matter and water bases. It was also asked whether high concentrations of Na^+^ and K^+^ salts have comparable effects on the growth of coastal *Rumex* species, and if this effect depends on the nature of anions used in treatment.

## 2. Materials and Methods

### 2.1. Plant Material

Seeds of *Rumex confertus* Willd. were collected in a mesophilic meadow near ponds in Salaspils, Latvia. Seeds of *Rumex hydrolapathum* Huds were collected in a seawater-affected wetland near Mērsrags, Latvia [3]. Seeds of *Rumex longifolius* DC. were collected on a shingle beach near Monti, Island of Saaremaa, Estonia. Seeds of *Rumex maritimus* L. were collected on a rocky shingle beach near Ohesaare, Island of Saaremaa, Estonia. Seeds were dried at room temperature for two weeks and stored at 4 °C until used.

### 2.2. Establishment, Cultivation and Treatments

Plants were cultivated in an experimental automated greenhouse during the autumn–winter season. Procedures used for plant establishment and cultivation were the same as described previously for *R. hydrolapathum* [21]. Briefly, seeds were germinated in autoclaved commercial garden soil (Biolan, Eura, Finland) in closed containers placed under periodic light and temperature conditions in a plant growth cabinet. Seedlings were individually transplanted to 200 mL plastic containers after appearance of the first two true leaves and gradually acclimated to greenhouse conditions (HortiMaX, Maasdijk, The Netherlands). Supplemented light was provided by Master SON-TPIA Green Power CG T 400W (Philips, Amsterdam, The Netherlands) and Powerstar HQI-BT 400 W/D PRO (Osram, Munich, Germany) lamps (photon flux density of photosynthetically active radiation 380 mol m^−2^ s^−1^ at the plant level), with a 16 h photoperiod. The day/night temperature was 23/16 C, and the relative air humidity was maintained at 60 to 70%. When seedlings reached 5–10 cm in height, they were individually transplanted to 1.2 L plastic containers filled with a 1.0 L mixture of garden soil and quartz sand (Saulkalne S, Saulkalne, Latvia) (1:3, *v*/*v*). Plants were watered with deionized water to keep soil moisture 60–70%. Plants were fertilized biweekly with Yara Tera Kristalon Blue fertilizer (19-6-20 + MgO + micro; Yara International, Oslo, Norway). A stock solution (125 g L^−1^) was made and plants were fertilized with 200 mL solution made from 5 mL of stock solution per L of deionized water.

Two weeks after transplantation, treatment with different salts started, with 5 individual plants per treatment. Each week, for 4 weeks, every individual plant received 1.0 g Na^+^ or 1.7 g K^+^ in the form of NaCl, KCl, NaNO_3_ or KNO_3_ until the final dose (4.0 g Na^+^ or 6.8 g K^+^) was reached. For NaNO_2_ and KNO_2_ a similar procedure was used, but due to the strongly negative effect of treatments (transient wilting of leaves after each treatment), the final concentration in soil was only 2.0 g Na^+^ and 3.4 g K^+^. Plants were cultivated for 7 more weeks after reaching the full treatment.

### 2.3. Plant Harvest and Measurements

Leaf chlorophyll concentration was measured one week before plant harvest using a chlorophyll meter CCM-300 (Opti-Sciences, Hudson, NH, USA). Three large actively photosynthesizing leaves from five plants per treatment were independently measured. Soil electrical conductivity (EC) in each individual plant container was measured at the end of the experiment in soil/deionized water extract (1: 50, *v*/*v*) using a LAQUAtwin conductivity meter B-771 (Horiba Scientific, Kyoto, Japan).

At harvest, plant leaves were separated according to their status and age/position as dry, large, middle, young and small (located in points of vegetative division of shoots). Leaves were counted and weighed separately. No “middle leaves” were designated for *R. hydrolapathum*. Plant roots were separated from soil and washed to remove any adhered particles. After drying at 60 °C for 72 h, dry mass was measured. The tissue water content was estimated as a mass of water in grams per gram of tissue dry mass.

For ion and EC analysis, plant tissues were crushed by hand to small pieces and a 0.2 g sample was randomly taken. Samples were ground to a fine powder using a mortar and pestle, and extracted with 10 mL deionized water for 1 min. After filtration through nylon mesh cloth (No. 80), concentrations of Na^+^, K^+^ and EC were measured by LAQUAtwin compact meters B-722, B-732 and B-771, respectively (Horiba Scientific, Kyoyo, Japan). Three samples of particular tissues representing individual plants were measured per treatment for each species in three analytical replicates. Ion concentration and EC were estimated on the basis of both dry biomass and tissue water.

### 2.4. Data Analysis

Results were analyzed by KaleidaGraph (v. 5.0, Synergy Software, Reading, PA, USA). Statistical significance of differences was evaluated by one-way ANOVA using post hoc analysis with minimum significant difference. Principal component analysis was performed by a freely available web program, ClustVis (http://biit.cs.ut.ee/clustvis/) (accessed on 30 November 2022) [28]. For graphs of principal component analysis, prediction ellipses were such that, with a probability of 0.95, a new observation from the same group will fall inside the ellipse. Unit variance scaling was applied to rows; singular value decomposition with imputation was used to calculate principal components.

## 3. Results

### 3.1. Morphological and Physiological Effects

**Figure 1:**
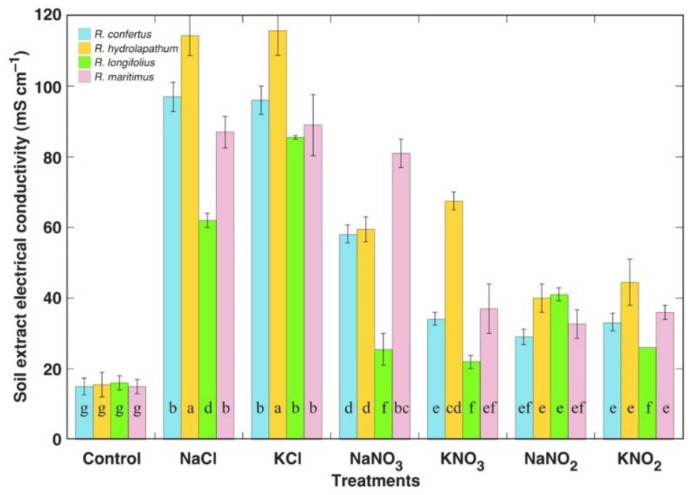
Effect of salinity treatments on soil extract electrical conductivity after cultivation of different *Rumex* species. Electrical conductivity was measure in 1:50 soil/water (*w*/*v*) extract. Data are means ± SE from 5 replicates. Different letters indicate statistically significant (*p* < 0.05) differences between treatments and species.

The remaining soluble salt concentration in substrate at the end of the experiment was evaluated by means of electrical conductivity analysis of soil water extracts (Figure 1). In general, the soil of plants treated with NaCl and KCl had the highest EC values. Lower levels in NaNO_3_ and KNO_3_ treatments reflected higher ion uptake due to higher plant biomass, but EC levels in NaNO_2_ and KNO_2_ treatments were lower mainly due to the fact that only half the amount of salts were used. Differences between the species for plants treated with the same type of salt most probably resulted from differences in biomass and ion uptake capacity.

**Figure 2:**
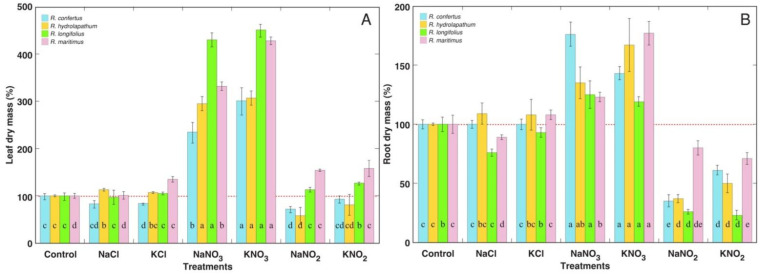
Relative effect of salinity treatments on leaf dry mass (**A**) and root dry mass (**B**) of different *Rumex* species. Data are means ± SE from 5 replicates. Different letters indicate statistically significant (*p* < 0.05) differences between treatments for a particular *Rumex* species.

**Figure 3:**
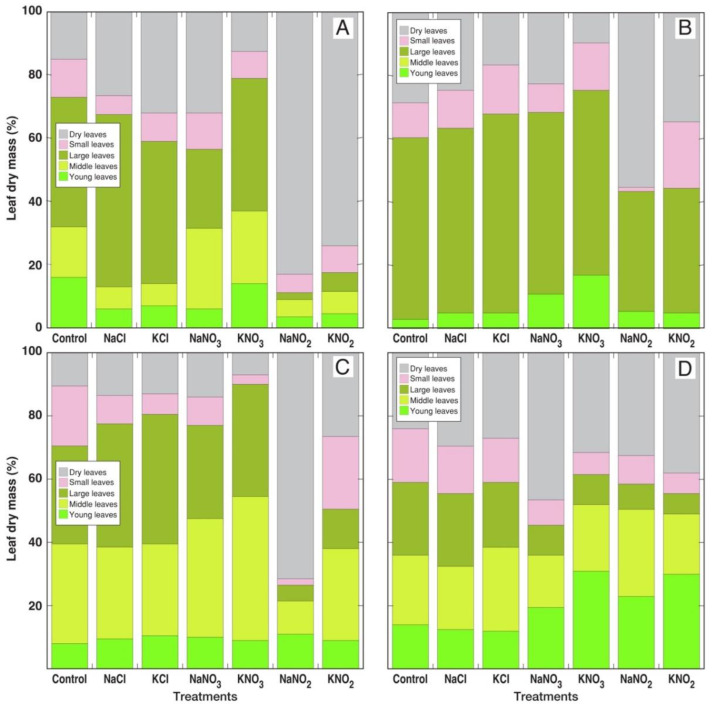
Relative changes in biomass partitioning in *Rumex confertus* (**A**), *Rumex hydrolapathum* (**B**), *Rumex longifolius* (**C**) and *Rumex maritimus* (**D**) plants under the effect of various salinity treatments.

General morphological effects of different salts on all *Rumex* species depended on their anion type, and were the same for specific Na^+^ and K^+^ salts (Figures S1–S4). Total dry mass of leaves was relatively little-affected by NaCl and KCl treatments (Figure 2A), but the relative contribution of different types of leaves in the biomass had been affected (Figure 3). Thus, contribution of dry leaves and large leaves increased and that of middle and young leaves decreased for *R. confertus* under chloride salinity (Figure 3A). However, the contribution of dry leaves decreased in chloride-treated *R. hydrolapathum* leaves (Figure 3B). Total leaf biomass significantly increased for all *Rumex* species in the case of treatment with nitrates (Figure 2A), but the contribution of dry leaves to biomass for KNO_3_-treated plants was lower than that for NaNO_3_-treated plants for all *Rumex* species (Figure 3). Contribution of dry leaves increased in NaNO_3_-treated *R. confertus* and *R. maritimus* plants in comparison to control, indicating their lower tolerance to this salt. Total biomass of leaves significantly decreased only in *R. confertus* and *R. hydrolapathum* plants under NaNO_2_ treatment, but it significantly increased in KNO_2_-treated *R. longifolius* plants and *R. maritimus* plants treated with both NaNO_2_ and KNO_2_ (Figure 2A). However, tolerance of *R. confertus* against nitrite salts was the lowest, as indicated by the extreme increase in contribution of dry leaves to total leaf biomass (Figure 3B). Both *R. hydrolapathum* and *R. longifolius* were more negatively affected by NaNO_2_ treatment in comparison to KNO_2_ treatment (Figure 3B,C). *R. maritimus* plants were the most tolerant to nitrite treatment, showing the least increase in the amount of dry leaf biomass (Figure 3D).

**Figure 4:**
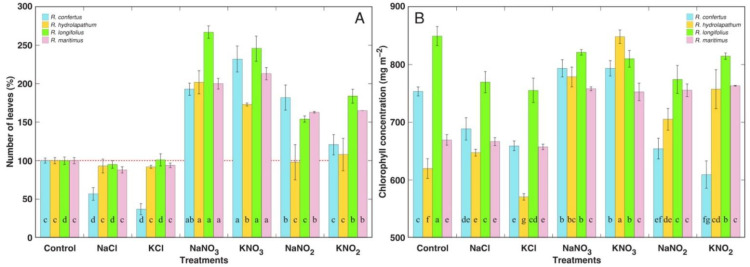
Relative changes in number of leaves (**A**) and changes in leaf chlorophyll concentration (**B**) under the effect of various salinity treatments in different *Rumex* species. Data are means ± SE from 5 replicates. For (**A**), different letters indicate statistically significant (*p* < 0.05) differences between treatments for a particular *Rumex* species. For (**B**), different letters indicate statistically significant (*p* < 0.05) differences between treatments and species.

Biomass of roots significantly decreased with chloride treatment only for *R. longifolius* and *R. maritimus* in the case of NaCl (Figure 2B). Treatment with nitrates resulted in a significant increase in root growth for all *Rumex* species. In contrast, treatment with nitrites resulted in a significant decrease in root growth for all *Rumex* species, with the relatively lowest effect evident in *R. maritimus*. Differences in the number of leaves between *Rumex* species and different treatments in general reflected their ability to maintain the formation of new leaves, even in a situation when rate of leaf senescence was increased in nitrite-treated plants (Figure 4A). However, for *R. confertus*, treatment with chlorides resulted in a significant decrease in leaf number.

Chlorophyll concentration in photosynthetically actively contributing leaves was a genotype-dependent characteristic (Figure 4). For the species with relatively high chlorophyll levels in control plants—*R. confertus* and *R. longifolius*—chlorophyll concentration significantly decreased under chloride treatment, with less pronounced changes under nitrate treatment. However, nitrate treatment had a pronounced stimulatory effect on chlorophyll concentration in *R. hydrolapathum* and *R. maritimus* plants. Nitrite treatment decreased chlorophyll concentration in *R. confertus* and *R. longifolius* plants, but increased it in *R. hydrolapayhum* and *R. maritimus* plants.

**Figure 5:**
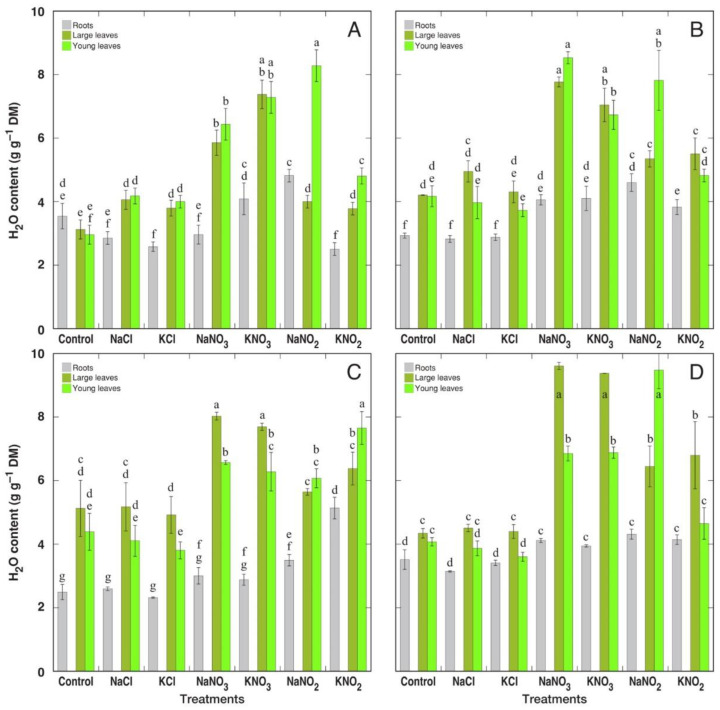
Changes in water content in different plant parts of *Rumex confertus* (**A**), *Rumex hydrolapathum* (**B**), *Rumex longifolius* (**C**) and *Rumex maritimus* (**D**) plants under the effect of various salinity treatments. Data are means ± SE from 5 replicates. Different letters indicate statistically significant (*p* < 0.05) differences between treatments for a particular *Rumex* species.

For *R. confertus,* water content significantly increased in leaves of plants treated with chlorides, and, to a larger extent, in nitrate-treated plants, as well as in young leaves of nitrite-treated plants (Figure 5A). For *R. hydrolpathum*, leaf water content significantly increased in plants treated with both nitrates and nitrites (Figure 5B). For *R. longifolius*, a significant increase in leaf water content was evident only for plants treated with nitrates (Figure 5C). For *R. maritimus*, leaf water content increased in both nitrate- and nitrite-treated plants (Figure 5D). Root water content was a relatively more stable parameter, but it significantly increased in *R. hydrolapathum* and *R. maritimus* plants treated with nitrates and nitrites as well as in nitrite-treated *R. longifolius* plants. Results from a statistical comparison of changes in water content in different plant parts under the effect of various treatments between the four *Rumex* species are shown in Table S1. Most importantly, it is evident that *R. maritimus* accumulated significantly more water in large leaves of nitrate-treated plants in comparison to other species.

Results of multivariate analysis showed that the morphological response of the four species to different salts was rather variable, with some larger similarity between *R. confertus* and *R. hydrolapathum* (Figure S5). Principal component analysis confirmed that, at the morphological level, Na^+^ and K^+^ salts with the same anion have similar effects (Figure S6A). In addition, by morphological responses, *R. maritimus* most prominently differed from *R. hydrolapathum* as well as *R. confertus* (Figure S6B).

### 3.2. Ion Accumulation

**Figure 6:**
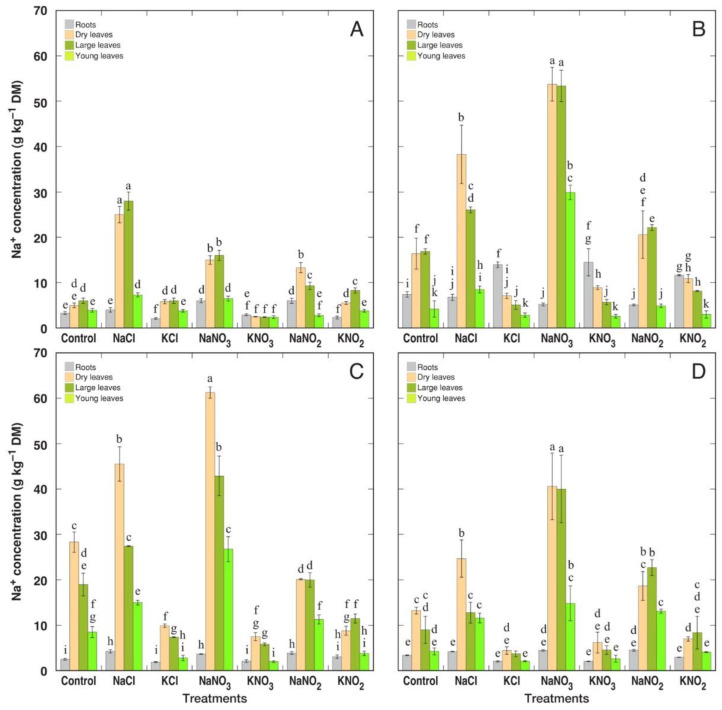
Changes in Na^+^ concentration on dry mass (DM) basis in different parts of *Rumex confertus* (**A**), *Rumex hydrolapathum* (**B**), *Rumex longifolius* (**C**) and *Rumex maritimus* (**D**) plants under the effect of various salts. Data are means ± SE from 3 replicates. Different letters indicate statistically significant (*p* < 0.05) differences between treatments for a particular *Rumex* species.

On a dry biomass basis, control plants of *R. hydrolapathum* and *R. longifolius* accumulated relatively high concentrations of Na^+^, reaching 30 g kg^−1^ for *R. longifolius* and 20 g kg^−1^ for *R. hydrolapathum* (Figure 6). *R. hydrolapathum*, *R. longifolius* and *R. maritimus* had the highest Na^+^ accumulation potential in NaNO_3_-treated plants, but *R. confertus* accumulated more Na^+^ in the case of NaCl treatment. The highest Na^+^ accumulation capacity was observed in *R. longifolius*, reaching 50 g kg^−1^ in NaCl-treated and 60 g kg^−1^ in NaNO_3_-treated plants (Figure 6C). Accumulation of Na^+^ in NaNO_2_-treated plants was similar for *R. hydrolapathum*, *R. longifolius* and *R. maritimus*, and lower for *R. confertus*. KCl and KNO_3_ treatment resulted in a significant decrease in Na^+^ concentration in leaves of *R. hydrolapathum*, *R. longifolius* and *R. maritimus* plants. For *R. confertus*, this effect was evident only for KNO_3_ treatment.

In general, young leaves accumulated less Na^+^ in comparison to older leaves, but the difference between large leaves and dry leaves was pronounced only in several treatments for *R. hydrolapathum*, *R. longifolius* and *R. maritimus* plants (Figure 6). Na^+^ content in roots was only slightly increased by treatment with Na^+^ salts and only for some species/treatment combinations.

**Figure 7:**
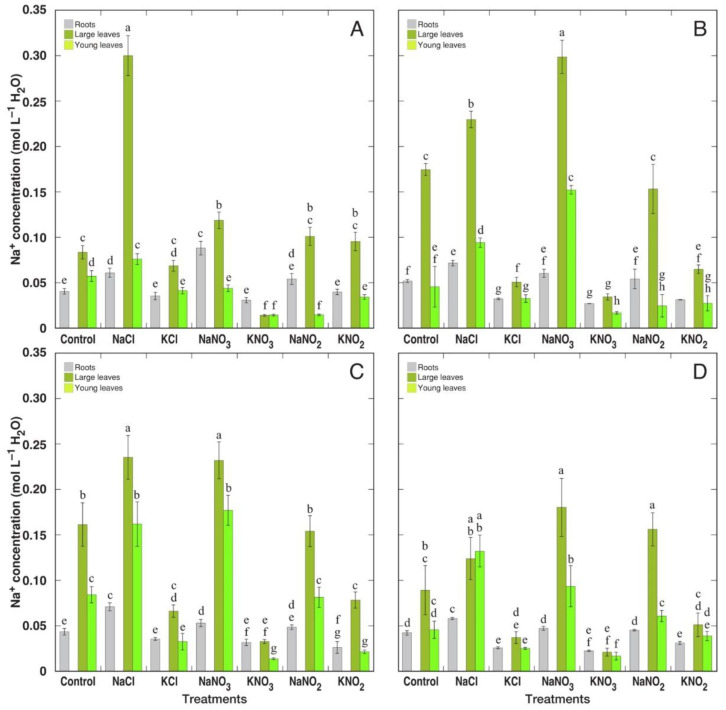
Changes in Na^+^ concentration on tissue water basis in different parts of *Rumex confertus* (**A**), *Rumex hydrolapathum* (**B**), *Rumex longifolius* (**C**) and *Rumex maritimus* (**D**) plants under the effect of various salts. Data are means ± SE from 3 replicates. Different letters indicate statistically significant (*p* < 0.05) differences between treatments for a particular *Rumex* species.

Due to differences in water content between particular *Rumex* species and in different treatments, Na^+^ concentration on a tissue water basis partially smoothed out the differences in Na^+^ content (Figure 7). *R. hydrolapathum* and *R. longifolius* plants still had the highest Na^+^ concentration in control conditions, but a relative increase in Na^+^ accumulation by treatment with Na^+^ salts was less pronounced. In addition, difference between NaCl and NaNO_3_ treatments diminished, except in *R. confertus* plants. All treatments with K^+^ salts resulted in a decrease in Na^+^ concentration in leaves of *R. hydrolapathum*, *R. longifolius* and *R. maritimus* plants, and only for KNO_2_ treatment for *R. confertus* plants.

**Figure 8:**
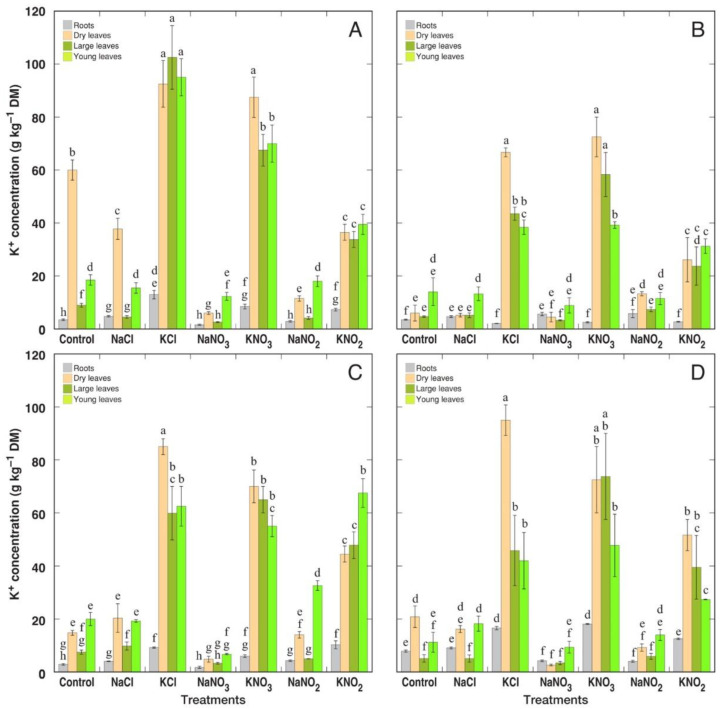
Changes in K^+^ concentration on dry mass (DM) basis in different parts of *Rumex confertus* (**A**), *Rumex hydrolapathum* (**B**), *Rumex longifolius* (**C**) and *Rumex maritimus* (**D**) plants under the effect of various salts. Data are means ± SE from 3 replicates. Different letters indicate statistically significant (*p* < 0.05) differences between treatments for a particular *Rumex* species.

*R. confertus* plants, treated with K^+^ salts, had the highest potential for K^+^ accumulation in comparison to other species (Figure 8). Variation in K^+^ concentrations between different treatments was relatively slightly pronounced. There was no consistently negative effect from treatment with Na^+^ salts on K^+^ concentration in plant tissues.

**Figure 9:**
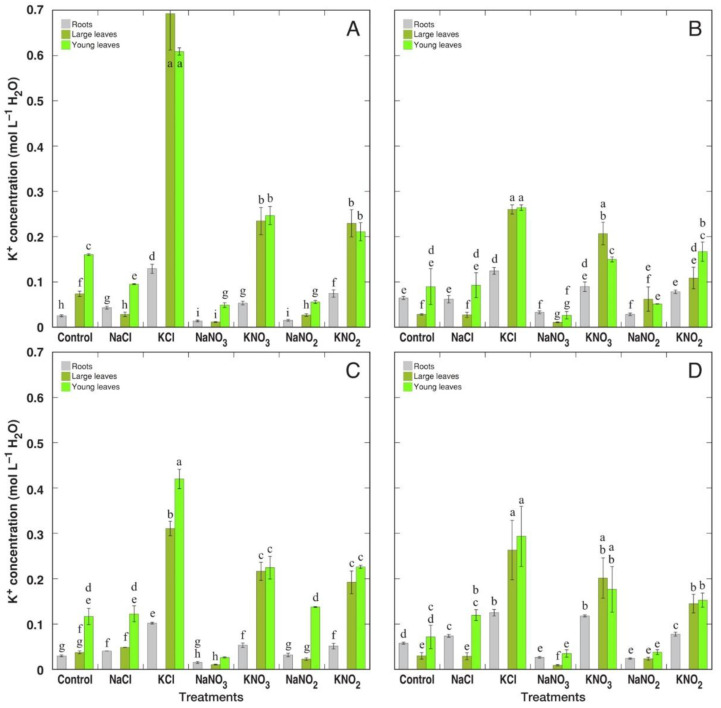
Changes in K^+^ concentration on tissue water basis in different parts of *Rumex confertus* (**A**), *Rumex hydrolapathum* (**B**), *Rumex longifolius* (**C**) and *Rumex maritimus* (**D**) plants under the effect of various salts. Data are means ± SE from 3 replicates. Different letters indicate statistically significant (*p* < 0.05) differences between treatments for a particular *Rumex* species.

When analyzed on tissue water basis, *R. confertus* plants treated with KCl showed the highest K^+^ accumulation potential (Figure 9A). For *R. longifolius* plants, KCl treatment also resulted in significantly higher K^+^ accumulation potential (Figure 9C). This effect was less pronounced for *R. hydrolapathum* (Figure 9B) and *R. maritimus* (Figure 9D) plants. The reducing effect of treatment with Na^+^ salts on K^+^ accumulation was significant for NaCl, NaNO_3_ and NaNO_2_ treatment of *R. confertus*; NaNO_3_ treatment of *R. hydrolapathum*; NaNO_3_ treatment of *R. longifolius*; as well as in large leaves of NaNO_3_-treated *R. maritimus* plants. There were no pronounced differences in K^+^ concentration between large and young leaves. In general, K^+^ accumulation potential for *Rumex* plants treated with K^+^ salts was higher than that for Na^+^ accumulation for plants treated with Na^+^ salts. Resulting values of the K^+^:Na^+^ molar concentration ratio in different parts of the four *Rumex* species are shown in Figure S7.

**Figure 10:**
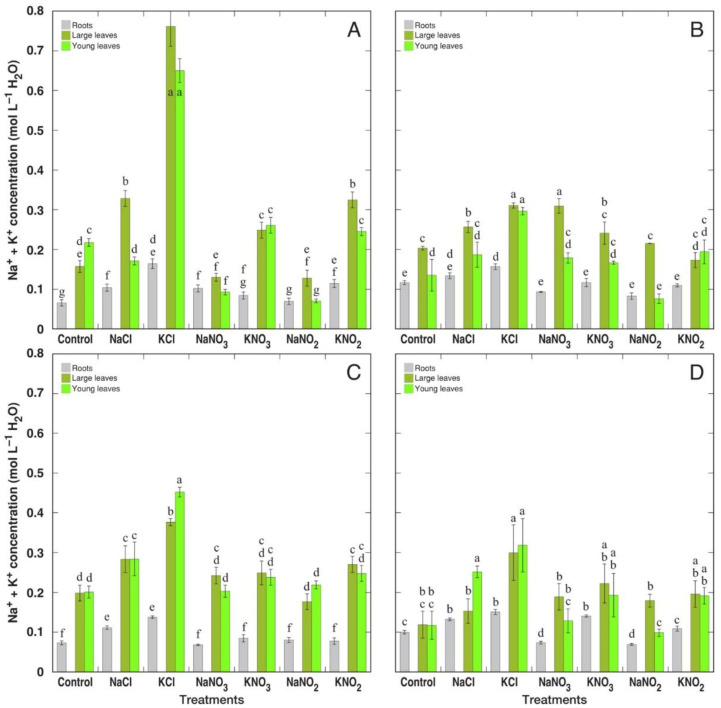
Changes in summed Na^+^ + K^+^ concentration on tissue water basis in different parts of *Rumex confertus* (**A**), *Rumex hydrolapathum* (**B**), *Rumex longifolius* (**C**) and *Rumex maritimus* (**D**) plants under the effect of various salts. Data are means ± SE from 3 replicates. Different letters indicate statistically significant (*p* < 0.05) differences between treatments for a particular *Rumex* species.

Values of summed Na^+^ + K^+^ concentration were relatively equalized (Figure 10), and salinity treatments resulted in relatively small increases in this parameter. The major exception was for *R. confertus* plants treated with KCl. Additionally, *R. longifolius* and *R. maritimus* plants showed the highest increase in Na^+^ + K^+^ concentration under KCl treatment. In some species/treatment combinations, large leaves had significantly higher Na^+^ + K^+^ concentration in comparison to young leaves.

**Figure 11:**
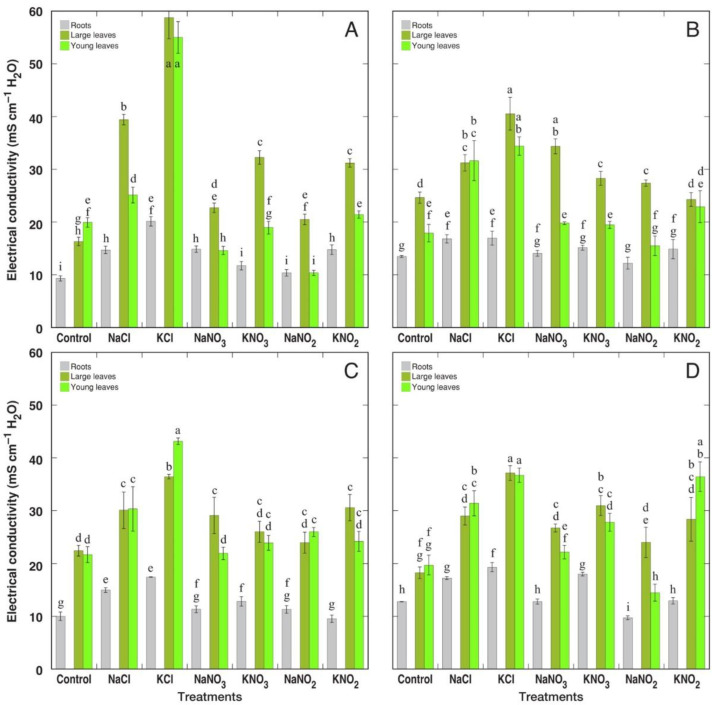
Changes in tissue electrical conductivity on tissue water basis in different parts of *Rumex confertus* (**A**), *Rumex hydrolapathum* (**B**), *Rumex longifolius* (**C**) and *Rumex maritimus* (**D**) plants under the effect of various salts. Data are means ± SE from 3 replicates. Different letters indicate statistically significant (*p* < 0.05) differences between treatments for a particular *Rumex* species.

Level of EC on a tissue water basis in general showed the same trend as that of summed Na^+^ + K^+^ concentration in tissue water (Figure 11). However, some inconsistencies were evident, equalization of variation between treatments and increased differences between large and young leaves, in some cases. Association between summed Na^+^ + K^+^ concentration and EC values in various plant parts of the four *Rumex* species was very tight (Figure S8). However, as *R. hydrolapathum* and *R. maritimus* plants showed relatively higher EC values at correspondingly lower Na^+^ + K^+^ concentration values in comparison to the other two species, it seems that *R. hydrolapathum* and *R. maritimus* plants accumulated additional electrolytically active substances besides Na^+^ and K^+^.

Principal component analysis revealed that the character of ion accumulation and EC in different parts of *Rumex* plants was significantly different for treatment with K^+^ salts, with no pronounced differences between KCl, KNO_3_ and KNO_2_ treatments (Figure S9A). However, treatments with NaCl, NaNO_3_ and NaNO_2_ resulted in relatively pronounced differences in respect to ion and electrolyte accumulation. There were no clearly distinguishable ion accumulation and EC value characteristics between the four *Rumex* species (Figures S9B and S10).

### 3.3. Overall Effect Assessment

To compare the overall effect of various Na^+^ and K^+^ salts on the four *Rumex* species, characteristics showing both major developmental (proportion of dry leaf biomass in total biomass of leaves and number of leaves) and physiological (water content, chlorophyll concentration and EC level in the main photosynthesizing leaves) adaptations with the most likely impact on overall plant performance over a long-term period were used (Table 1). Comparing the effects of Na^+^ and K^+^ in chloride-treated plants, it is evident that K^+^ resulted in a higher increase in both the proportion of dry leaves and EC levels in large leaves. Additionally, the number of leaves of *R. confertus* decreased more under KCl treatment in comparison to NaCl treatment. Developmental effects of Na^+^ and K^+^ in the form of nitrates was species-specific, but NaNO_3_ was more unfavorable for *R. confertus* and *R. maritimus* in comparison to KNO_3_ and less favorable for *R. hydrolapathum* and *R. longifolius*. For nitrite salts, NaNO_2_ treatment resulted in a higher proportion of dry leaf biomass for *R. confertus*, *R. hydrolapathum* and *R. longifolius* in comparison to KNO_2_ treatment, but the opposite effect was evident for *R. maritimus.* General similarities and differences in major effects between cation/anion combinations for various salts were also confirmed by principal component analysis (Figure S11). Effects of NaCl and KCl were relatively similar, but more differences were seen between NaNO_3_ and KNO_3_ and between NaNO_2_ and KNO_2_.

The results of multivariate analysis showed that the overall developmental and physiological responses of *R. hydrolapathum* and *R. maritimus* plants to salinity were relatively more similar, and were markedly different from the other two species, which formed separate clusters each (Figure S12).

## 4. Discussion

In the present study, we tried to look at the problem of Na^+^ accumulation in plants growing on saline substrates with a broader perspective, considering both Na^+^ and K^+^ as electrolytically active species contributing to the ionic strength of cellular environment [4]. Based on the theory of halophytic plants either as includers or excluders of Na^+^, it seemed to be useful to compare coastal origin accessions of different taxonomically related plant species in respect to their Na^+^ and K^+^ accumulation potential when growing in a substrate with an increased concentration of either element in association with different anions. Similar to the recent previous studies showing that a number of relatively salinity-tolerant plant species display highly comparable responses to both Na^+^ and K^+^ chloride, the same response was found also for three *Rumex* species with accessions from coastal habitats (*R. hydrolapathum*, *R. longifolius*, *R. maritimus*) as well as the cosmopolitan species *R. confertus*.

It is usually thought that Na^+^ as a metal has special characteristics, leading to its significant toxicity in comparison to K^+^. However, chemical features of the two elements are relatively similar. The main difference between Na^+^ and K^+^ is associated with the different hydration ability of the ions: while K^+^ as a large ion has only a weak hydration ability, Na^+^ as a smaller ion has relatively strong hydration potential and is capable of tightly attaching 0.25 water molecules [29]. This results in a weak chaotropic activity of K^+^ in contrast to Na^+^, which can be considered weakly kosmotropic. It appears also that Na^+^ has higher potential biological activity as a ligand for monovalent cation-dependent enzyme anion intermediates with similar water affinity [30]. There are significant physiological differences related to the different roles of the two elements in plants. The majority of cellular K^+^ is accumulated in the vacuole, contributing to osmotic potential in the form of simple salts for plants in non-saline soils [31]. While cytosolic concentration of K^+^ is relatively tightly regulated at 100 to 200 mmol L^−1^, K^+^ concentration in the vacuole is rather variable [31]. In addition, Na^+^ accumulation in salt-tolerant plants is thought to represent a mechanism of osmotic adjustment [2]. In this situation, the majority of Na^+^ is sequestered in the vacuole together with low amounts of K^+^ as a part of a mechanism for tissue salinity tolerance, but K^+^ is retained in cytosol in order to sustain cellular activity, including photosynthesis [32].

Besides osmotic adjustment, other functional characteristics of the two ions are of interest. Thus, electrolytic activity is a main characteristic of both elements. Cellular processes, such as enzyme activation, are greatly affected by electrostatic interactions due to the presence of ions, characterized by ionic strength [33]. Electrical conductivity can be used as an indirect indication of ionic strength in a cellular solution, as ionic strength is a function of ion concentration and electric charge. This approach has been approved in simpler systems, such as soil solution [34]. K^+^ appears to be the main constituent of ionic strength in plants necessary for enzyme activation, as this ion can constitute 10% on a dry mass basis [31].

The common concept in respect to the K^+^/Na^+^ relationship for plants in saline conditions is that, with an inevitable increase in cytosolic Na^+^ concentration, the concentration of K^+^ in cytoplasm decreases, and that the ability of halophytes to maintain a high K^+^:Na^+^ ratio determines their salinity tolerance [35]. However, the idea that, at least in some salt-tolerant species, Na^+^ can functionally replace K^+^ has also been experimentally assessed. Thus, by comparing the effects of different Na^+^ and K^+^ salts on growth and physiological changes in the subtropical coastal species *Sesuvium portulacastrum*, it was found that the positive effect of Na^+^ salts was significantly higher than that of K^+^ salts, but acetate salts of both cations as well as KCl resulted in severe growth inhibition [10]. In the present study, all *Rumex* species were able to maintain high biomass accumulation rates irrespective of their internal K^+^:Na^+^ molar concentration ratio, as evident for plants treated with NaNO_3_.

Comparing negative aspects of Na^+^ vs. K^+^ on plant growth, the number of leaves was more severely reduced in *R. confertus* plants by KCl in comparison to NaCl (Figure 4), but leaf dry mass was lower for *R. maritimus* plants treated with NaNO_2_ in comparison to KNO_2_ (Figure 2A). Leaf senescence was more pronounced in all *Rumex* species treated with NaNO_3_ in comparison to KNO_3_ and in *R. hydrolapathum* and *R. longifolius* plants treated with NaNO_2_ in comparison to KNO_2_ (Figure 3). Additionally, root growth was more inhibited in *R. longifolius* and *R. maritimus* plants treated with NaCl in comparison to KCl and in *R. confertus* and *R. hydrolapathum* plants treated with NaNO_2_ in comparison to KNO_2_ (Figure 2B). However, no significant differences were found in respect to the positive effects of Na^+^ vs. K^+^ on plant growth in the case of stimulation of leaf development and dry mass accumulation by nitrates.

Certain differences in plant responses to Na^+^ and K^+^ have also been noted in other studies. For *Salicornia europaea*, K^+^ salts had more negative effects on plant growth in comparison to respective Na^+^ salts [36]. Similarly, high concentrations of KCl had more pronounced inhibitory effects on the growth of *Atriplex nummularia* plants in comparison to identical concentrations of NaCl [9]. For a salt-resistant accession of *Chenopodium album*, KCl at 300 mM inhibited plant growth more significantly in comparison to NaCl at the same concentration [37]. Additionally, the growth of plants from a coastal accession of *Armeria maritima* was more negatively affected by KCl treatment in comparison to that of NaCl, and no induction of non-ionic osmolyte accumulation occurred in KCl-treated plants [11]. In contrast, the growth of *Mentha aquatica* plants was more unfavorably affected by treatment with NaCl in comparison to KCl [12].

It was concluded that both Na^+^ and K^+^ equally contribute to cellular osmotic adjustment in conditions of high soil salinity for several halophyte species [9,38]. Moreover, when taxonomically related genotypes were compared, it was suggested that Na^+^ vs. K^+^ accumulation capacity depends on whether the particular accession is adapted either to saline or xeric conditions, with K^+^ accumulation capacity prevailing in plants adapted under low soil-moisture conditions [38]. In the present study, the only non-wetland species, *R. confertus*, accumulated a significantly higher concentration of K^+^ under KCl supply in comparison to wetland-adapted species (Figure 9A), which accounted also for the highest tissue EC level on a water basis (Figure 11A). However, similar differences were not seen in *R. confertus* plants treated with K^+^ in the form of nitrate or nitrite.

In the natural conditions of salt-affected coastal habitats, the Na^+^ accumulation capacity of *R. maritimus* was within the middle 50% of values among means from all analyzed species, but it was relatively higher than that for *R. hydrolapathum* and *R. longifolius* [4]. When expressed on a tissue water basis, variations in Na^+^ concentration decreased for all three species. This coincides with the results of the present study, showing that Na^+^ accumulation potential for *R. hydrolapathum* and *R. longifolius* was higher than that for *R. maritimus*, and differences between various salts smoothed out in respect to Na^+^ concentration in tissue water (Figure 6 and Figure 7). The three *Rumex* species in natural conditions were not among the highly electrolyte-accumulating species, and based on intraspecific variability of Na^+^ and K^+^ concentration, *R. hydrolapathum* was classified as a tight EC regulating plant [4]. However, this was not fully evidenced by the results of the present study. While Na^+^ concentration indeed decreased in plants treated with K^+^-containing salts (Figure 6), a similar decrease in K^+^ concentration in Na^+^-treated plants was seen only for some species/salt combinations (Figure 8).

Similarity in effects between Na^+^ and K^+^ could be related to the fact that surplus Na^+^ and K^+^ have similar fates (including mechanisms of uptake, translocation and compartmentation) in relatively well salt-tolerant species. However, differences between various anions are most likely related to differences in physiological functions and metabolic fates of particular ions. It is evident from the other studies that the form of anion in Na^+^ compounds does affect their action on plants. In some cases, Na^+^ in the form of sulfate is shown to be more toxic than that in the form of chloride [39]. Na^+^ and K^+^ in the form of chloride had the least effect on the growth parameters of *Rumex* species. The number of leaves was negatively affected only for *R. confertus* (Figure 4A), along with increased intensity of older leaf senescence (Figure 3A). In addition, root growth was significantly inhibited by NaCl treatment for *R. longifolius* and *R. maritimus* (Figure 2B).

Entirely positive effects of either Na^+^ and K^+^ nitrates on both shoot and root growth for all four *Rumex* species could be related to the fact that these species can be characterized as nitrophilous, with elevated requirements for nitrogen availability to sustain maximum growth. Indeed, several wetland *Rumex* species are known as highly nitrogen-demanding [40]. Sodium nitrate-dependent growth stimulation has been described also for other salt-tolerant coastal species, i.e., *Salicornia europaea* [17] and *Sesuvium portulacastrum* [10].

The most important physiological effect of nitrate—and, in part, also nitrite—treatment found in the present study was a significant increase in leaf water content for all *Rumex* species (Figure 5). As a result, electrolyte concentrations on a tissue water basis in leaves of nitrate- and nitrite-treated plants were significantly lower than that in chloride-treated or control plants (Figure 10). This finding somehow contradicts the widespread opinion that a Na^+^ dilution mechanism is a characteristic response of salt-adapted accumulating species to increasing salinity [41,42]. Rather, at least in the particular case, this points to a positive relationship between tissue water content and plant nitrogen nutrition status, as shown for other experimental systems [43,44].

Nitrite is especially available in aquatic environments where it accumulates as a result of ammonia oxidation at an elevated pH or in flooded soil at a lower pH in oxygen-shortage conditions [45]. Nitrite is toxic for plants, mostly because of the formation of NO [46], but it is usually rapidly reduced to NH_4_^+^ by nitrite reductase and can accumulate only in tissues deficient in reduction capacity [47]. Nitrite treatment usually results in reduced plant growth and mineral deficiency [48,49], but nitrite has been indicated as an important source of N [47], especially in conditions not favoring nitrous acid production in substrate [50]. The toxicity threshold of nitrite in soil is in a range 75–225 mg kg^−1^ [51]. Many wetland plants are highly tolerant to an increased concentration of nitrogen forms, including nitrite [52]. In the present study, total nitrite concentration reached 4 g L^−1^ substrate in a treatment with NaNO_2_ or KNO_2_. Rate of older leaf senescence significantly increased for *R. confertus* plants with both Na^+^ and K^+^ nitrites (Figure 3A), and for *R. hydrolapathum* and *R. longifolius* plants with Na^+^ nitrite (Figure 3B,C), but not for *R. maritimus* plants (Figure 3D). However, root growth was significantly inhibited for all species by nitrite treatments, with the least degree of inhibition in *R. maritimus* (Figure 2B), suggesting that nitrite reduction in roots can indeed serve as an important tolerance mechanism against nitrite toxicity [47]. NaNO_2_ had a more pronounced negative effect in comparison to that of KNO_2_ or NaCl, suggesting synergistic negative effect of Na^+^ and NO_2_^–^ on plant growth.

A comparison of the effect of salinity on the four species showed that the result of the comparison was significantly dependent on the parameters used. Analysis of morphological parameters revealed similarity between *R. confertus* and *R. hydrolapathum* (Figure S5), which is not surprising since both these species also show some morphological similarity under control conditions. However, characteristics important for adaptation at the level of developmental and physiological parameters showed similarity between *R. hydrolapathum* and *R. maritimus* (Figure S12). This similarity points to the fact that *R. maritimus* most likely is a coastal-specific species in the Baltic region [24], but the particular accession of *R. hydrolapathum* from a seawater-affected coastal marsh represents a possible coastal ecotype of the species [3]. Interestingly, analysis of ion accumulation and EC levels in different plant parts showed that each species has a very unique response to salinity (Figure S10), further supporting the idea that a particular pattern of ion accumulation in salt-tolerant species is not directly related to their salinity tolerance. However, for *R. confertus*—generally not occurring in salt-affected habitats [22]—increased electrolyte accumulation in KCl-treated plants in comparison to NaCl-treated ones was associated with the more unfavorable effect of KCl, seen as increased biomass of senescent leaves and reduced leaf number (Table 1).

## 5. Conclusions

Salinity tolerance of all coastal-associated accessions of *Rumex* species was high, but the inland species *R. confertus* was the least tolerant to salinity. Similarity in effects between Na^+^ and K^+^ could be related to the fact that surplus Na^+^ and K^+^ have similar fates (including mechanisms of uptake, translocation and compartmentation) in relatively well salt-tolerant species. However, differences between various anions are most likely related to differences in physiological functions and metabolic fates of particular ions. The coastal-specific species *R. maritimus* showed exceptional tolerance to nitrite treatment. It is evident that coastal *Rumex* species can be used in further studies aiming at understanding their salinity tolerance mechanisms at both physiological and molecular levels.

## Figures and Tables

**Table 1:** Changes in developmental and physiological parameters of different *Rumex* species due to treatment with various salts (% increase or decrease in respect to control plants is shown).

Species	Salt	Proportion of Dry Leaves in Leaf Biomass (%)	Number of Leaves (%)	H_2_O Content in Large Leaves (%)	Chlorophyll Concentration in Large Leaves (%)	EC in Large Leaves (%)
*R. confertus*	NaCl	+77	−43	+30	−9	+142
	KCl	+113	−63	+21	−12	+260
	NaNO_3_	+113	+93	+88	+5	+39
	KNO_3_	0	+132	+137	+5	+98
	NaNO_2_	+453	+82	+28	−13	+26
	KNO_2_	+393	0	+21	−19	+91
*R. hydrolapathum*	NaCl	0	0	0	+4	+27
	KCl	+43	0	0	−8	+64
	NaNO_3_	−22	+102	+85	+26	+39
	KNO_3_	−67	+73	+68	+36	+15
	NaNO_2_	+90	0	+27	+14	+11
	KNO_2_	0	0	+31	+22	0
*R. longifolius*	NaCl	0	0	0	−9	+34
	KCl	0	0	0	−11	+54
	NaNO_3_	0	+167	+57	−3	+30
	KNO_3_	−33	+146	+50	−5	0
	NaNO_2_	+581	+54	0	+14	0
	KNO_2_	+152	+84	0	+22	+36
*R. maritimus*	NaCl	0	0	0	0	+59
	KCl	0	0	0	0	+103
	NaNO_3_	+94	+100	+121	+13	+46
	KNO_3_	+31	+113	+116	+13	+70
	NaNO_2_	+35	+63	+49	+13	+31
	KNO_2_	+58	+65	+57	+14	+55

Only statistically significant changes (*p* < 0.05) are taken into account.

## Data Availability

All data reported here is available from the authors upon request.

## Supplementary Materials

The following supporting information can be downloaded at: https://www.mdpi.com/article/10.3390/plants12010092/s1, Table S1: Results of statistical comparison of changes in water content in different plant parts under the effect of various treatments between the model species. Figure S1: Typical *Rumex confertus* plants during the experiment *Rumex confertus* plants during the experiment; Figure S2: Typical *Rumex hydrolapathum* plants during the experiment; Figure S3: Typical *Rumex lomgifolius* plants during the experiment; Figure S4: Typical *Rumex maritimus* plants during the experiment; Figure S5: Generated heat map and cluster analysis on effect of various salts on number of leaves, dry mass of leaves and roots of different *Rumex* species. Figure S6: Principal component analysis on effect of different salts on number of leaves, dry mass of leaves and roots of different *Rumex* species. Figure S7: Changes in K^+^:Na^+^ molar concentration ratio in different parts of *Rumex confertus* (A), *Rumex hydrolapathum* (B), *Rumex longifolius* (C), and *Rumex maritimus* (D) plants under the effect of various salts; Figure S8: Relationship between Na^+^ + K^+^ concentration and electrical conductivity in different parts of *Rumex confertus* (A), *Rumex hydrolapathum* (B), *Rumex longifolius* (C), and *Rumex maritimus* (D) plants under the effect of various salts; Figure S9: Principal component analysis on effect of various salts on accumulation of Na^+^ and K^+^, and electrical conductivity in different parts of different *Rumex* species. Figure S10: Generated heat map and cluster analysis on accumulation of Na^+^ and K^+^, and electrical conductivity in different parts of different *Rumex* species; Figure S11: Principal component analysis on overall developmental and physiological effect of various salts in different *Rumex* species; Figure S12: Generated heat map and cluster analysis on overall developmental and physiological effect of various salts in different *Rumex* species.
