# Supplementary material for: Type of Anion Largely Determines Salinity Tolerance in Four Rumex Species
Source: Plants (Basel). 2022 Dec 24;12(1):92. doi: 10.3390/plants12010092 (PMC9823408; doi:10.3390/plants12010092)
Supplement: Supplementary file 1 [file plants-12-00092-s001.zip › plants-2058680-supplementary.pdf]

Type of Anion Largely Determines Salinity Tolerance in Four *Rumex* Species

Zaiga Landorfa-Svalbe, Una Andersone-Ozola and Gederts Ievinsh

Table S1. Results of statistical comparison of changes in water content in different plant parts under the effect of various treatments between the model species. Values are presented in Figure 5. Different letters indicate statistically significant ($p < 0.05$) differences between *Rumex* species for a particular treatment.

Treatment	*R. confertus*	*R. hydrolapathum*	*R. longifolius*	*R. maritimus*
Roots				
Control	a	a	a	a
NaCl	ab	b	b	a
KCl	bc	b	c	a
$NaNO_3$	bc	a	b	a
KNO_3	a	a	b	a
$NaNO_2$	a	a	b	a
KNO_2	c	b	a	b
Large leaves				
Control	a	a	a	a
NaCl	a	a	a	a
KCl	a	a	a	a
$NaNO_3$	d	c	b	a
KNO_3	b	b	b	a
$NaNO_2$	b	a	a	a
KNO_2	b	a	a	a
Young leaves				
Control	b	a	a	a
NaCl	a	a	a	a
KCl	a	a	a	a
$NaNO_3$	b	a	b	b
KNO_3	a	a	a	a
$NaNO_2$	a	ab	b	a
KNO_2	b	b	a	b

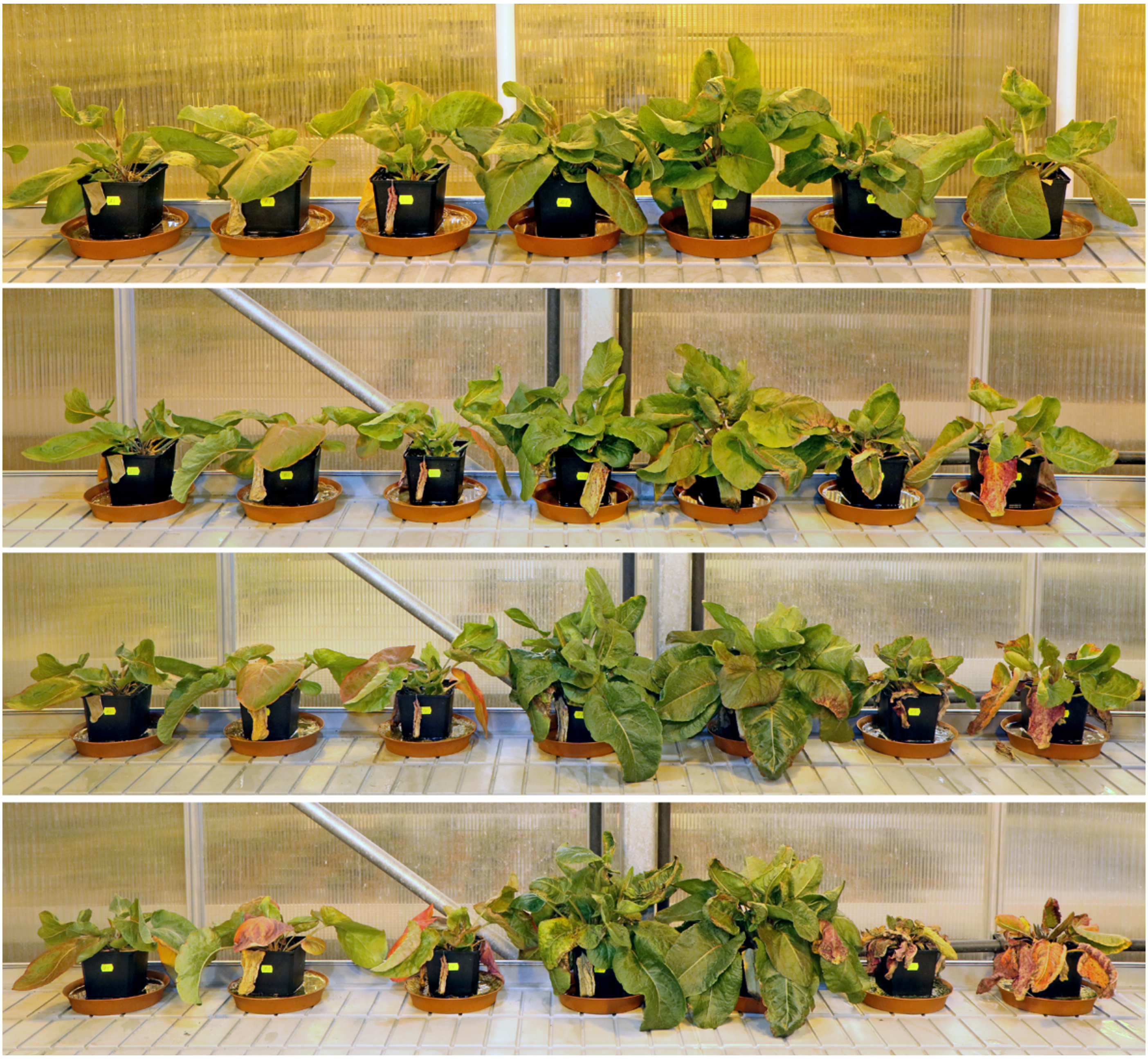

Figure S1. Typical *Rumex confertus* plants during the experiment. From top to bottom: 1, 3, 5, 7 weeks after full treatment. From left to right: control, NaCl, KCl, $NaNO_3$, KNO_3, $NaNO_2$, KNO_2. NaCl and $NaNO_3$ treatments contained 4.0 g L^{-1} Na^+, KCl and KNO_3 treatments contained 6.8 g L^{-1} K^+, $NaNO_2$ treatment contained 2.0 g L^{-1} Na^+, KNO_2 treatment contained 3.4 g L^{-1} K^+.

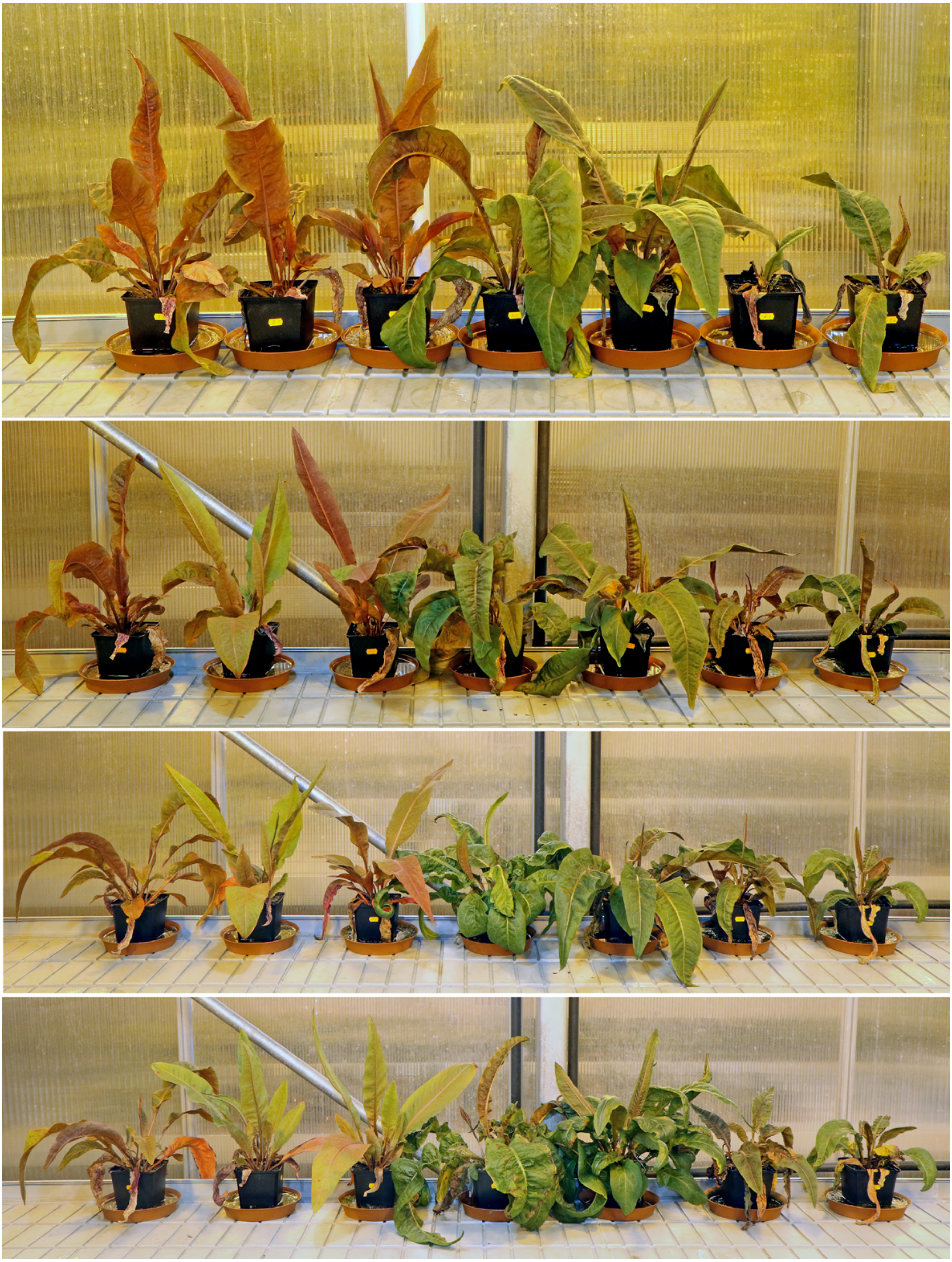

Figure S2. Typical *Rumex hydrolapathum* plants during the experiment. From top to bottom: 1, 3, 5, 7 weeks after full treatment. From left to right: control, NaCl, KCl, $NaNO_3$, KNO_3, $NaNO_2$, KNO_2. NaCl and $NaNO_3$ treatments contained 4.0 g L^{-1} Na^+, KCl and KNO_3 treatments contained 6.8 g L^{-1} K^+, $NaNO_2$ treatment contained 2.0 g L^{-1} Na^+, KNO_2 treatment contained 3.4 g L^{-1} K^+.

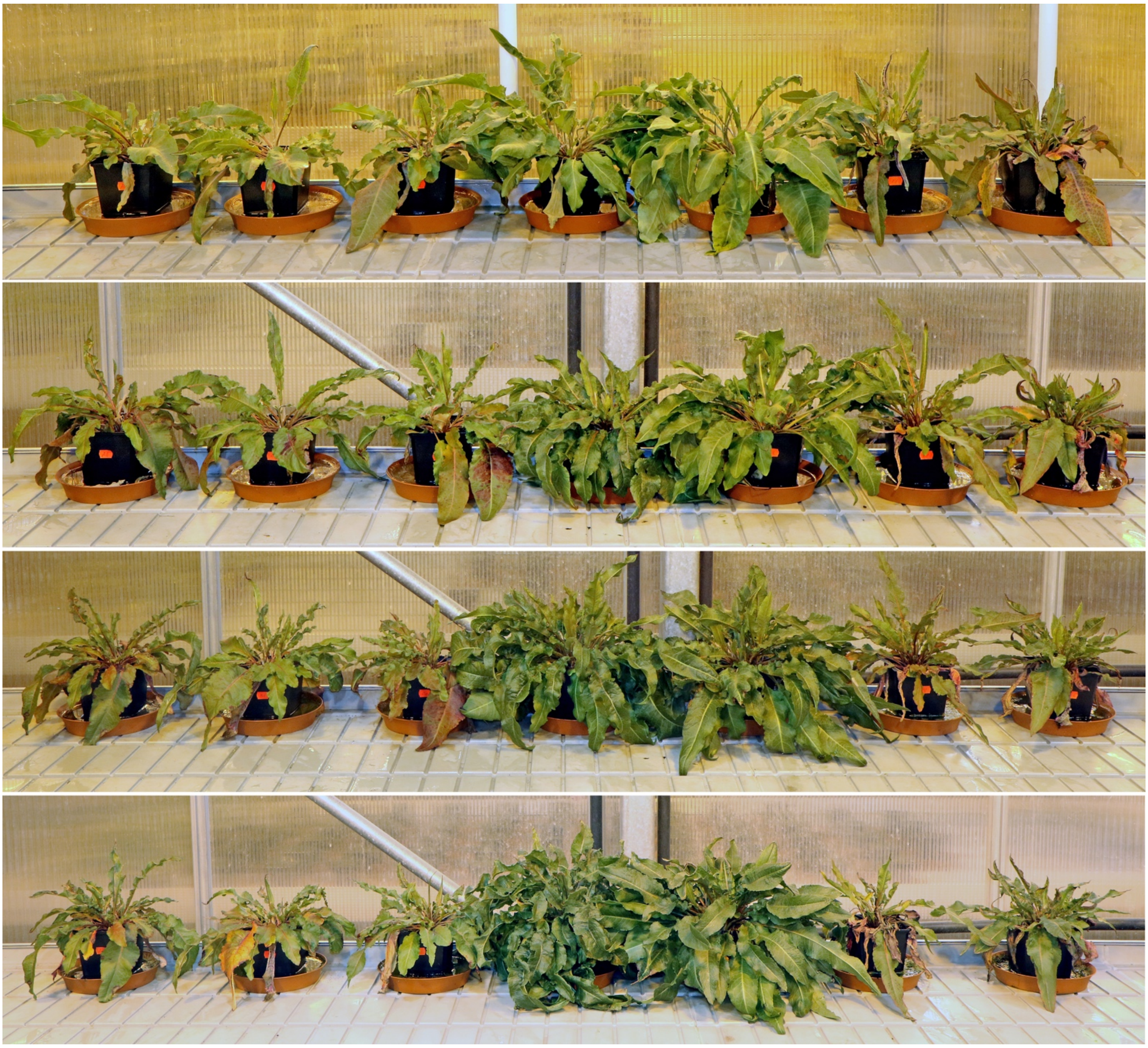

Figure S3. Typical *Rumex lomgifolius* plants during the experiment. From top to bottom: 1, 3, 5, 7 weeks after full treatment. From left to right: control, NaCl, KCl, $NaNO_3$, KNO_3, $NaNO_2$, KNO_2. NaCl and $NaNO_3$ treatments contained 4.0 g L^{-1} Na^+, KCl and KNO_3 treatments contained 6.8 g L^{-1} K^+, $NaNO_2$ treatment contained 2.0 g L^{-1} Na^+, KNO_2 treatment contained 3.4 g L^{-1} K^+.

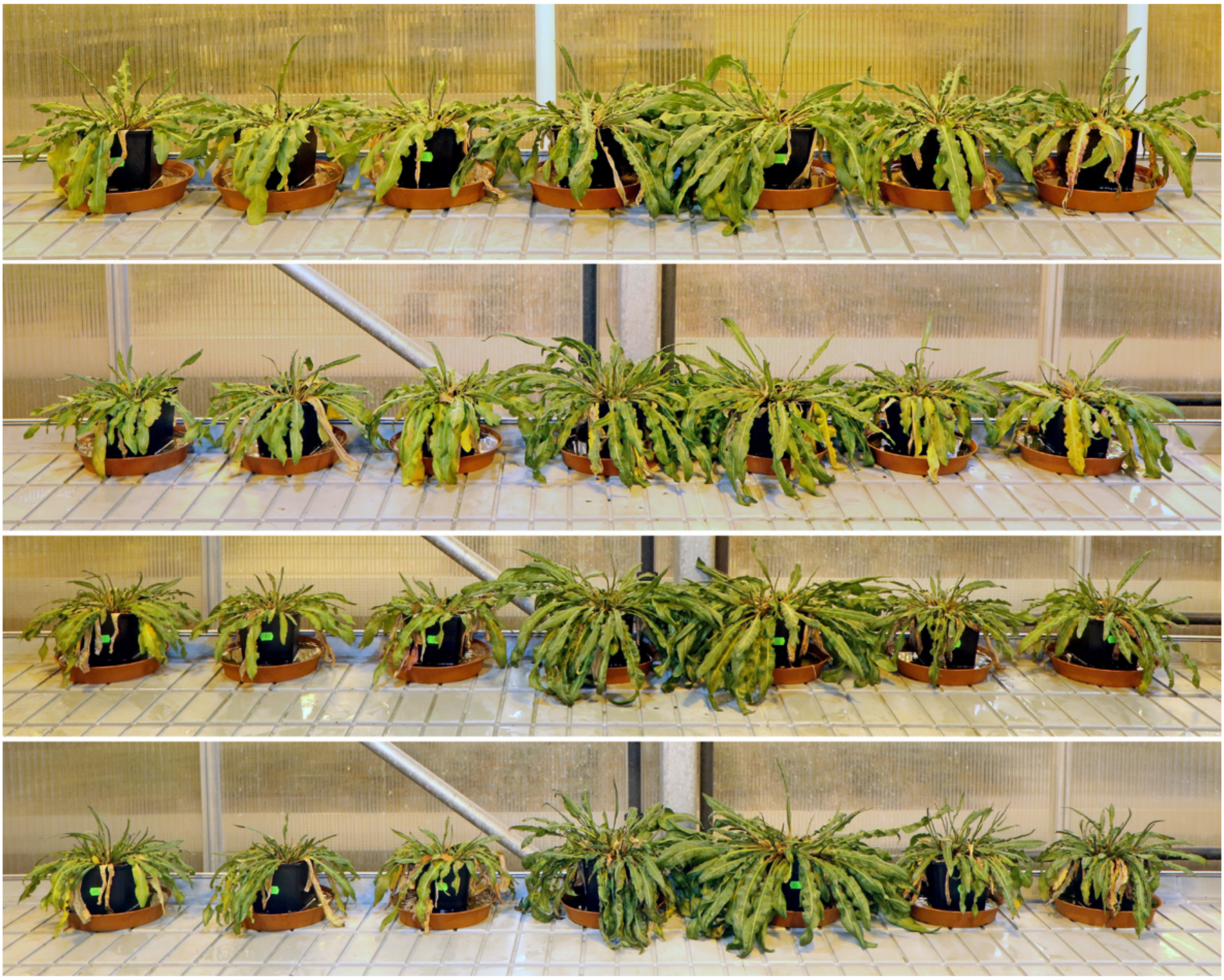

Figure S4. Typical *Rumex maritimus* plants during the experiment. From top to bottom: 1, 3, 5, 7 weeks after full treatment. From left to right: control, NaCl, KCl, $NaNO_3$, KNO_3, $NaNO_2$, KNO_2. NaCl and $NaNO_3$ treatments contained 4.0 g L^{-1} Na^+, KCl and KNO_3 treatments contained 6.8 g L^{-1} K^+, $NaNO_2$ treatment contained 2.0 g L^{-1} Na^+, KNO_2 treatment contained 3.4 g L^{-1} K^+.

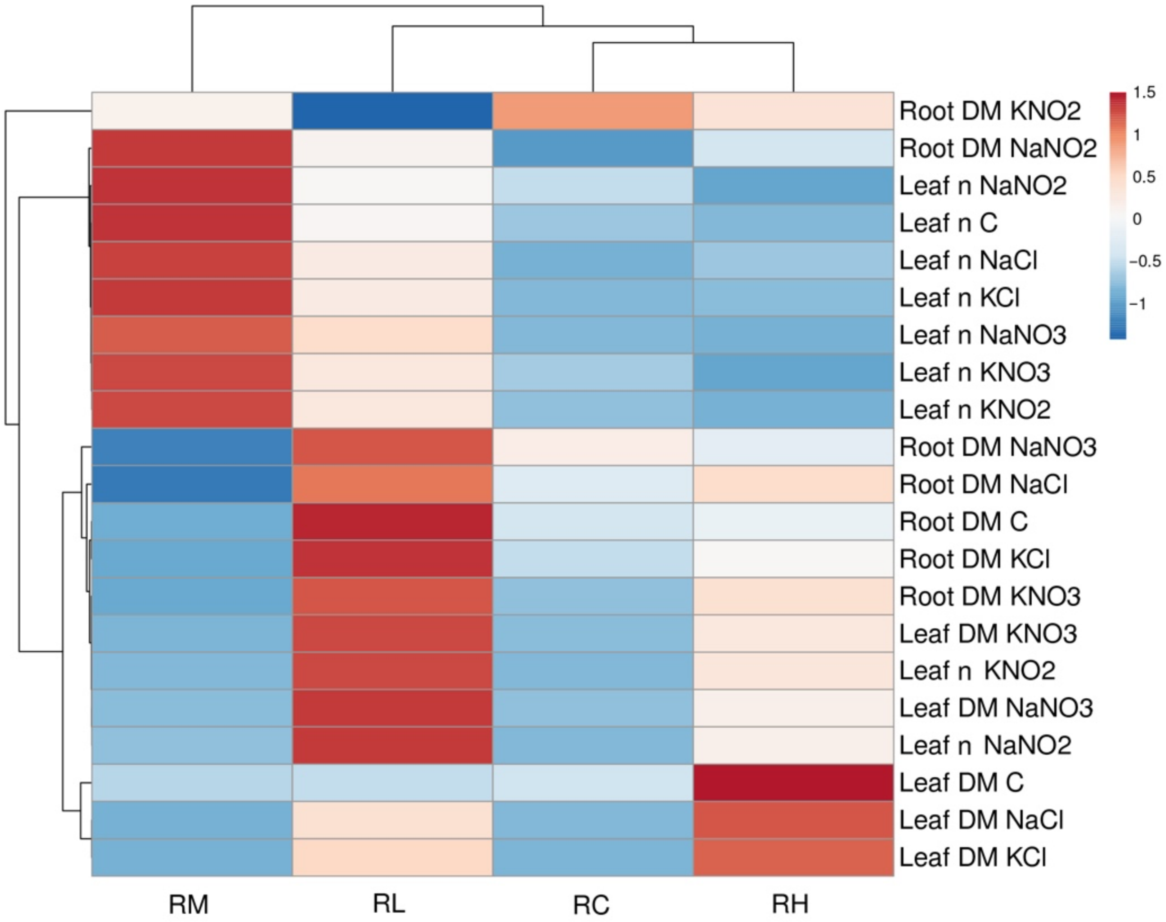

Figure S5. Generated heat map and cluster analysis on effect of various salts on number of leaves, dry mass of leaves and roots of different *Rumex* species. Hierarchical clusters were generated by average linkage method with correlation distance. Color scale shows relative intensity of normalized parameter values. RH, *Rumex hydrolapathum*, RC, *Rumex confertus*; RL, *Rumex longifolius*; RM, *Rumex maritimus*; DM, dry mass; n, number; C, control.

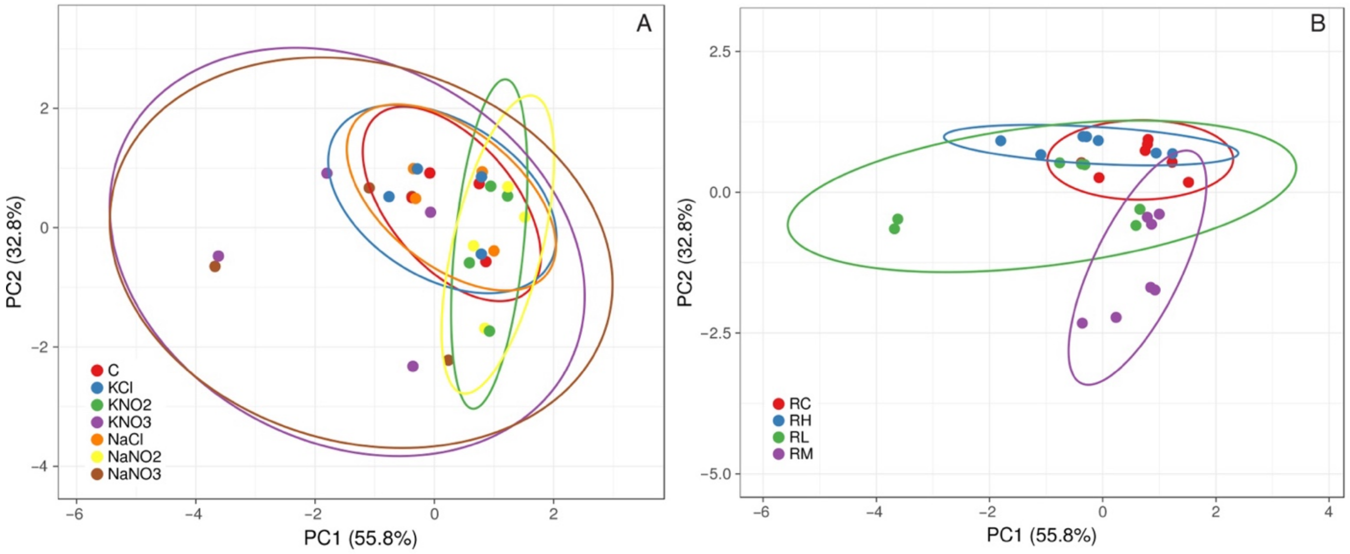

Figure S6. Principal component analysis on effect of different salts on number of leaves, dry mass of leaves and roots of different *Rumex* species. A, grouping according different treatments by species; B, grouping according *Rumex* species by treatments. Prediction ellipses are such that with probability 0.95, a new observation from the same group will fall inside the ellipse. Unit variance scaling was applied to rows; singular value decomposition with imputation was used to calculate principal components. X and Y axis show principal component 1 and principal component 2 that explain 55.8% and 32.8% of the total variance, respectively. RH, *Rumex hydrolapathum*, RC, *Rumex confertus*; RL, *Rumex longifolius*; RM, *Rumex maritimus*.

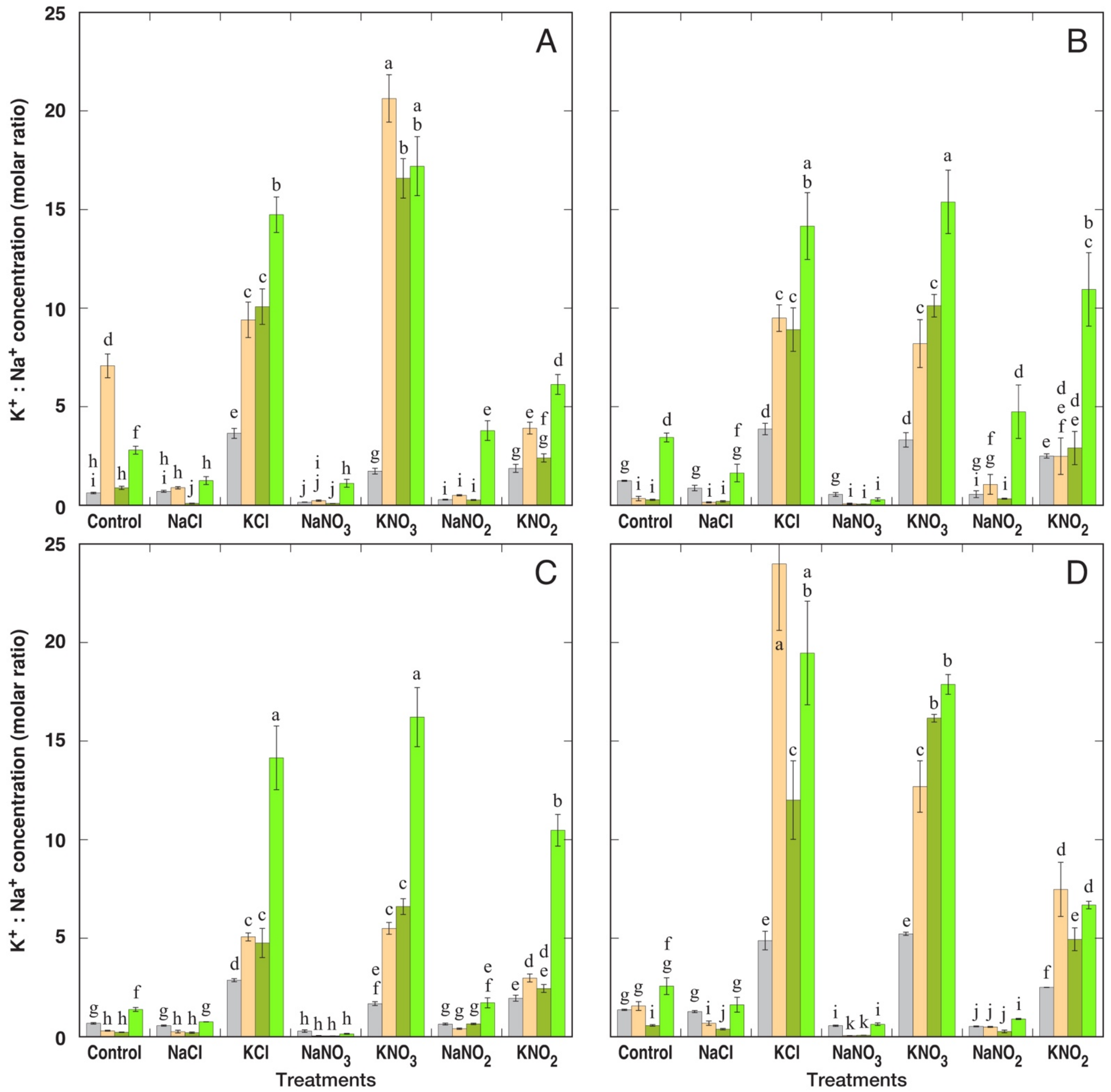

Figure S7. Changes in K^+ : Na^+ molar concentration ratio in different parts of *Rumex confertus* (A), *Rumex hydrolapathum* (B), *Rumex longifolius* (C), and *Rumex maritimus* (D) plants under the effect of various salts. Data are means ± SE from 3 replicates. Different letters indicate statistically significant ($p < 0.05$) differences between treatments for a particular *Rumex* species.

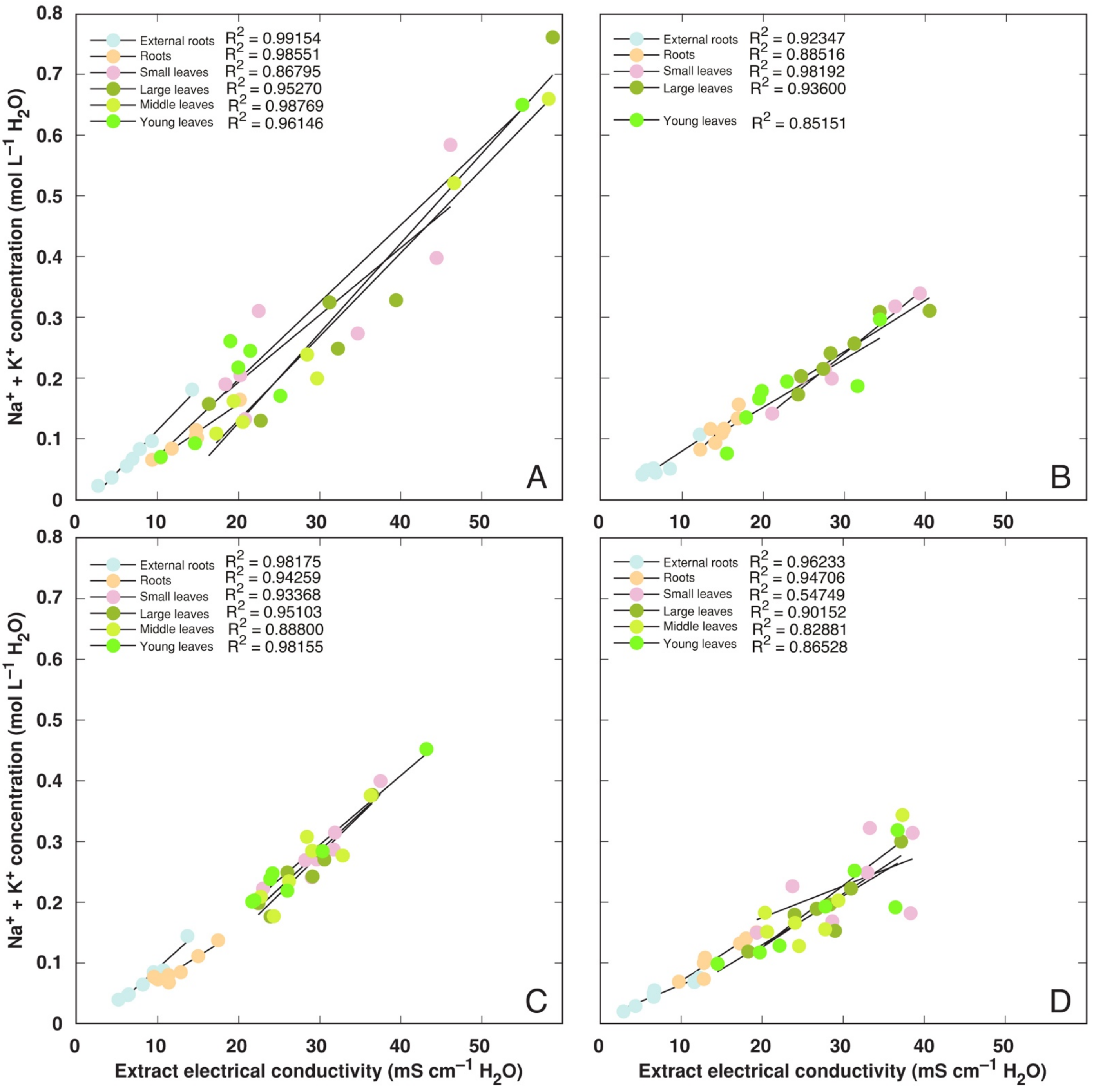

Figure S8. Relationship between Na^+ + K^+ concentration and electrical conductivity in different parts of *Rumex confertus* (A), *Rumex hydrolapathum* (B), *Rumex longifolius* (C), and *Rumex maritimus* (D) plants under the effect of various salts.

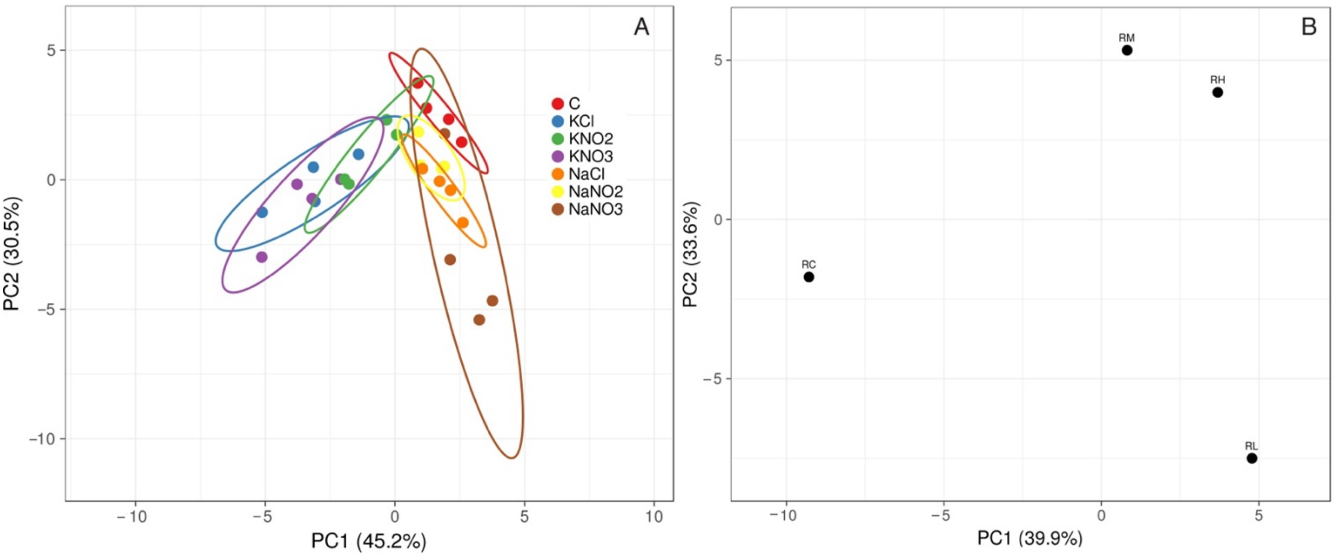

Figure S9. Principal component analysis on effect of various salts on accumulation of Na^+ and K^+, and electrical conductivity in different parts of different *Rumex* species. A, grouping according different treatments; B, grouping according to *Rumex* species. Prediction ellipses are such that with probability 0.95, a new observation from the same group will fall inside the ellipse. Unit variance scaling was applied to rows; singular value decomposition with imputation was used to calculate principal components. RH, *Rumex hydrolapathum*, RC, *Rumex confertus*; RL, *Rumex longifolius*; RM, *Rumex maritimus*.

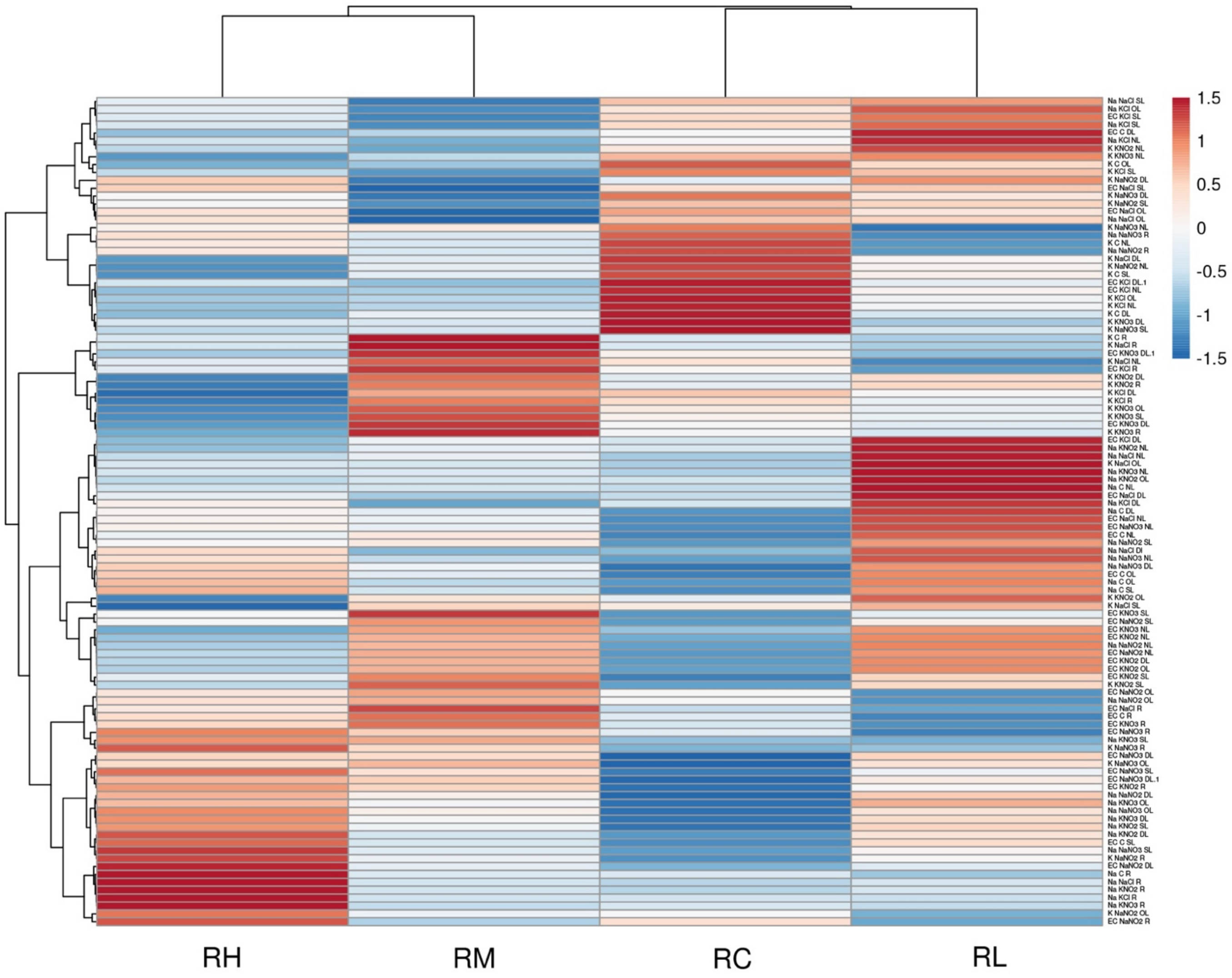

Figure S10. Generated heat map and cluster analysis on accumulation of Na^+ and K^+, and electrical conductivity in different parts of different *Rumex* species. Hierarchical clusters were generated by average linkage method with correlation distance. Color scale shows relative intensity of normalized parameter values. RH, *Rumex hydrolapathum*, RC, *Rumex confertus*; RL, *Rumex longifolius*; RM, *Rumex maritimus*.

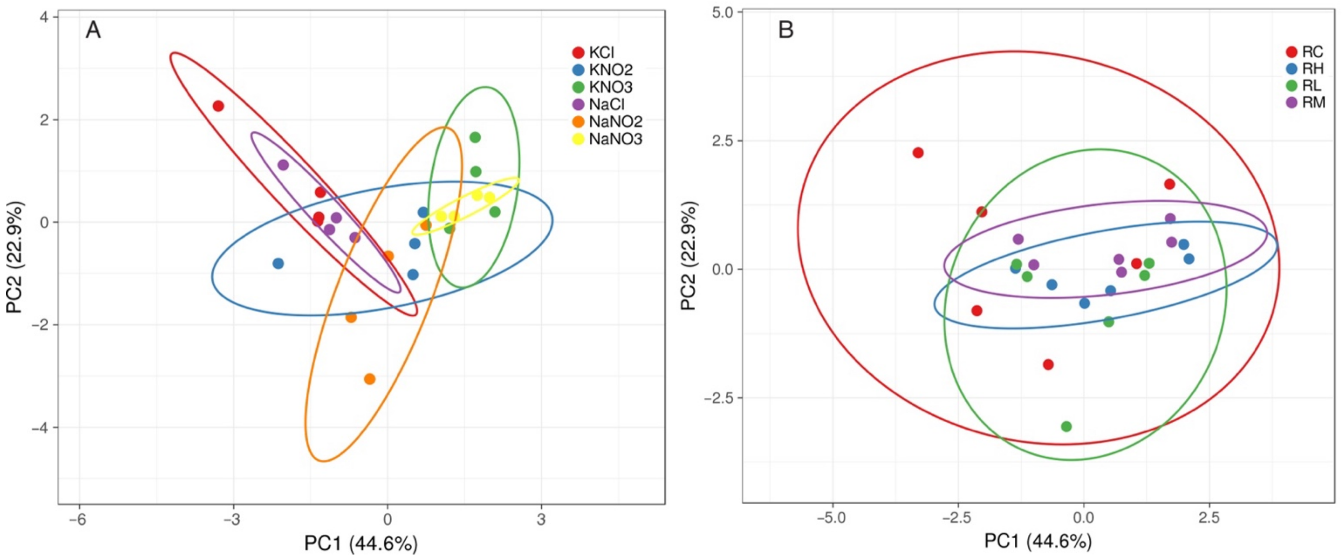

Figure S11. Principal component analysis on overall developmental and physiological effect of various salts in different *Rumex* species. A, grouping according different treatments; B, grouping according *Rumex* species. Prediction ellipses are such that with probability 0.95, a new observation from the same group will fall inside the ellipse. Unit variance scaling was applied to rows; singular value decomposition with imputation was used to calculate principal components. RH, *Rumex hydrolapathum*, RC, *Rumex confertus*; RL, *Rumex longifolius*; RM, *Rumex maritimus*.

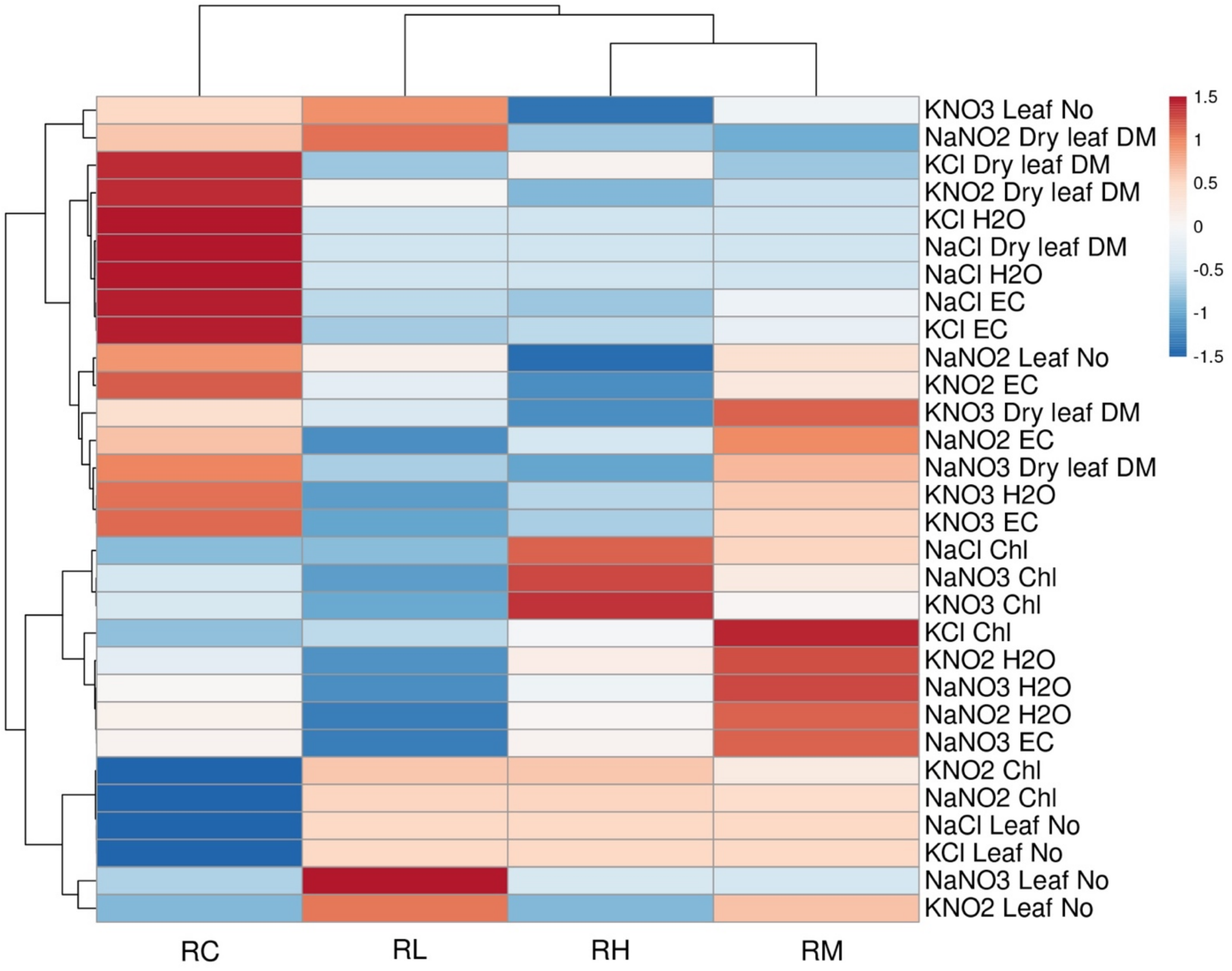

Figure S12. Generated heat map and cluster analysis on overall developmental and physiological effect of various salts in different *Rumex* species. Hierarchical clusters were generated by average linkage method with correlation distance. Color scale shows relative intensity of normalized parameter values. RH, *Rumex hydrolapathum*, RC, *Rumex confertus*; RL, *Rumex longifolius*; RM, *Rumex maritimus*; Leaf No, total number of leaves; Dry leaf DM, relative proportion of dry leaves in total biomass of leaves; EC, electrical conductivity on tissue water basis; Chl, leaf chlorophyll concentration.
